# A machine vision based defect detection method for coated carbide CNC inserts and its industrial automation implementation analysis

**DOI:** 10.1038/s41598-026-52293-1

**Published:** 2026-05-11

**Authors:** Junqi Hu, Shi Chen, Sheng Yin, Song Qiu

**Affiliations:** 1 School of Physical Science and Technology, Southwest Jiaotong University, Chengdu, 610031 China; 2 CENTECH-EG Co., Ltd., Chengdu, China

**Keywords:** CNC machine tools, Deep learning, Metal surface defect detection, Small defect detection, Engineering, Mathematics and computing

## Abstract

Computer Numerical Control (CNC) inserts are critical components of CNC machine tools, where surface defects can severely compromise machining precision. Traditional manual inspection methods for these defects are inefficient and prone to significant oversight. To address these limitations, this paper presents an automated real-time system for detecting surface defects on inserts. A dedicated dataset of CNC tool inserts was created and annotated with defect categories. We propose an Attention-Augmented Multi-Defect YOLO model (A2MD-YOLO) for surface defect detection on CNC inserts. The development of this model is motivated by key characteristics of the dataset, which include substantial variation in defect sizes, high intra-class appearance variance, and low inter-class variance. A2MD-YOLO achieves higher detection efficiency and accuracy while reducing the rate of missed detections. The A2MD-YOLO model demonstrates a substantial performance improvement, with the $mAP_{50-95}$ increasing from 0.529 to 0.571 and the missed detection rate decreasing from 21.4% to 11.8%. Finally, the proposed algorithm was implemented into the hardware system, enabling automated detection of surface defects on CNC inserts.

## Introduction

CNC (Computer Numerical Control) machine tools, known as the “mother machines” of modern industry, integrate computerized numerical control technology with computer-aided design and manufacturing into traditional machining processes. They consist of several key components, including the CNC unit, servo systems, machine tool body, and measurement feedback devices. Compared with conventional machine tools, CNC machine tools offer marked improvements in dimensional accuracy, repeatability, and production efficiency. They also feature enhanced capabilities for manufacturing complex products, higher automation levels, and reduced changeover times. As a result, they are widely employed in critical sectors such as aerospace, automotive, and medical device manufacturing^1^.

**Fig. 1:**
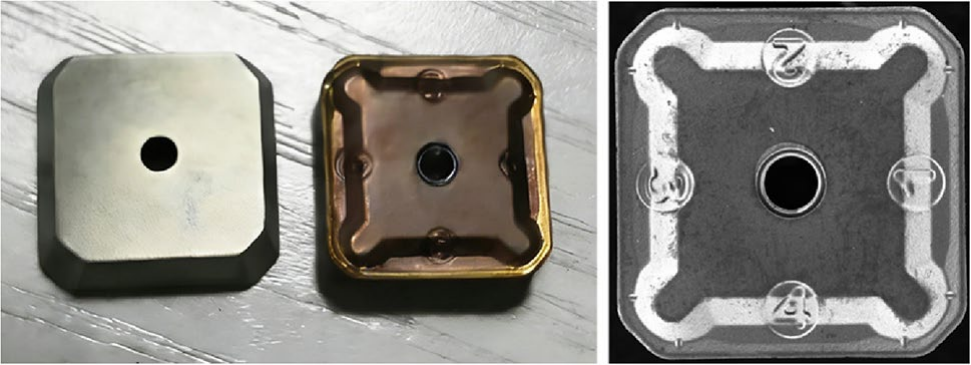
Coated cemented carbide CNC insert sample.

The high precision and efficiency of CNC machine tools are attributed to their sophisticated mechanical structures, advanced CNC systems, and servo drive technologies. However, the high-level capabilities of these hardware and software systems must ultimately be realized through the interaction between the cutting execution unit–the CNC tooling system–and the workpiece^2,3^. With ongoing industrial transformation and upgrading, market demands for the performance of machine tools and cutting tools are continuously increasing. High-performance precision indexable CNC tools are rapidly replacing traditional cutting tools, representing a clear development trend. Among these, CNC tool inserts, as the terminal functional components directly responsible for material removal, play a decisive role in determining the maximum potential of CNC machine tools and the actual machining outcomes^4^. Key performance indicators of CNC tool inserts include wear resistance, toughness, chemical stability, and chip-breaking capability. These properties are determined by controllable factors such as insert material and geometric design, and can be continuously improved through technological advancements. However, during manufacturing and transportation, uncontrollable factors such as impact, corrosion, and variations in processing techniques can introduce surface defects of varying shapes and sizes on the inserts. These surface imperfections significantly impair service life and cutting accuracy. Therefore, surface defect inspection of CNC tool inserts is an indispensable step after manufacturing and before their deployment in machining operations.

Currently, the detection of surface defects on inserts still predominantly relies on manual visual inspection in many industrial settings. However, there is a scarcity of academic research focused on defect detection specifically within the domain of CNC inserts. Li et al.^5^ developed a deep anomaly detection method (CNN-AD) using spindle current signals to predict tool breakage with high accuracy, though it cannot classify or locate defects and may miss failures without significant load fluctuations. Zhang et al.^6^ applied an improved YOLOv3 algorithm to localize and measure tool spiral edge defects via image processing, yet the method is geometry-specific and not transferable to CNC inserts. Zeng et al.^7^ enhanced YOLOv5 to automatically detect surface defects in CBN inserts with 90% accuracy and real-time speed, although detection of minute defects remains limited.

While the aforementioned studies have contributed to advancing defect detection in CNC tool inserts, this research direction continues to face several significant challenges. Firstly, the substantial production demands in modern industrial machining impose stringent requirements for mechanized and automated quality inspection of CNC tool inserts, necessitating highly efficient detection systems. Secondly, unlike common computer vision tasks that benefit from benchmark datasets such as ImageNet and COCO–containing hundreds of thousands to millions of images–there is no universal dataset available for CNC tool insert defect detection. Existing datasets are typically tailored for specific applications and remain largely inaccessible to the public^8^. Lastly, due to influences from background environments, illumination intensity, and defect scale, the detection of such defects imposes exceptionally high demands on model performance. To address the limitation of current manual visual inspection for surface defect detection on CNC inserts, this paper explores the application of machine vision to replicate the human inspection process, aiming to develop an automated solution that is real-time, accurate, and efficient. Figure 1 illustrates a cemented carbide CNC insert with coating, which is utilized in our work as a case. Given that CNC inserts are predominantly manufactured from hard metal materials such as cemented carbide and cubic boron nitride (CBN), studies conducted by scholars on surface defects in metallic materials can provide valuable references for defect detection in CNC inserts.

### Related works

#### Non-deep learning detection methods

In the field of metal surface defect detection, traditional approaches have long relied on manual visual inspection. This method is not only labor-intensive but also highly dependent on the inspector’s experience and subjective judgment, leading to inconsistent outcomes. When dealing with large-volume production, minuscule defect sizes, or low optical contrast, the rates of missed and false detections rise significantly^8,9^. Subsequently, image processing techniques combined with traditional machine learning models gained popularity in surface defect inspection.Research specifically targeting CNC insert surface defects is scarce; however, this study focuses on coated carbide inserts, which differ in material properties, optical characteristics, and defect morphology from other insert types. Given that CNC inserts are metal components, their surface defect detection can be positioned within the broader field of metal surface defect detection, allowing us to draw upon relevant methodological references. Gyimah et al.^10^ propose a robust framework combining a Non-Local Means filter with Completed Local Binary Pattern (RCLBP) for accurate surface defect detection under noisy and varying conditions. Tang et al.^11^ developed an image-processing-based system capable of real-time, high-precision identification of solder joint and thread damage defects, though such methods remain limited by reliance on manual feature analysis and extraction^12,13^. Hong et al.^14^ proposed a HOG + SVM method for detecting coating shedding damage in underwater pipelines using ultrasonic imaging. While demonstrating the applicability of traditional methods to image-based defect detection, this approach relies on manually designed features (HOG) and detects only a single defect type, limiting its scalability to multi-class classification tasks. Kundu et al.^15^ employed a random forest regression approach for predicting the remaining useful life of spur gears subjected to pitting failure, demonstrating the effectiveness of ensemble decision trees in handling vibration-based health indicators. However, such methods are designed for online prognostics and cannot provide the fine-grained localization and classification of surface defects that offline vision-based inspection achieves. Liu et al.^16^ proposed a method based on improved K-nearest neighbor (KNN) and Euclidean clustering segmentation for detecting surface defects on lithium batteries using 3D point cloud data. By introducing voxel density and point cloud discreteness parameters, their approach effectively identifies defects such as bubbles, folds, warping, and pits. This study further demonstrates the versatility of traditional machine learning methods in metal surface defect detection across different imaging modalities. Subsequently, non-destructive testing techniques including ultrasonic, eddy-current, and magnetic flux leakage have been widely adopted for metal surface inspection^17^. Jiang et al.^18^ proposed a laser ultrasonic surface wave method for quantitative detection of rail surface cracks, establishing a non-contact system to assess cracks of different depths. Peng et al.^19^ developed a dynamic scanning platform integrating an optical rail mover and an ACFM probe to address signal distortion in ACFM-based high-speed rail crack detection. Yet, these NDT methods often require sophisticated equipment and specific environmental conditions, making them less suitable for real-time, high-throughput manufacturing environments. Such constraints hinder their application in highly automated production lines where speed, efficiency, and adaptability are critical^8^.

#### Deep learning-based detection methods

In recent years, deep learning models have been extensively applied in the domain of object detection^20,21^, achieving remarkable results and becoming a vital tool for enhancing automation in detection processes. These models are capable of learning complex patterns and features directly from data through multi-layer neural networks, enabling accurate identification of various object types. Models employed for object detection algorithms are primarily categorized into two types: two-stage models and one-stage models. Representative two-stage models (based on convolutional neural networks) include Fast R-CNN, Faster R-CNN, and Mask R-CNN. Prominent one-stage models encompass the You Only Look Once (YOLO) algorithm and the Single Shot Multibox Detector (SSD) algorithm. The YOLO series of single-stage object detectors has undergone continuous evolution in both architecture and performance. YOLOv5 utilized an anchor-based design strategy, whereas YOLOv8 introduced an anchor-free detection head and established a unified multi-task architecture capable of simultaneously handling object detection, classification, instance segmentation, and pose estimation, significantly improving both the versatility and accuracy of the model. Subsequent versions, including YOLOv11 and YOLOv12, further concentrated on structural optimizations, such as enhancing the backbone network, refining feature fusion modules, and integrating attention mechanisms, thereby strengthening feature modeling capability and improving small object detection performance. The latest version, YOLO26, adopted strategies including the removal of the Distribution Focal Loss module and the incorporation of the Multi-Gradient Stochastic Gradient Descent optimizer. These modifications not only led to improved accuracy in small object detection but also achieved approximately 43% faster inference speed on CPU devices. YOLOv8 was released in 2023, it effectively balances speed and accuracy, enabling real-time, high-precision detection tasks, and has consequently been widely adopted in industrial applications^22,23^. However, the YOLO series also exhibits certain limitations, such as suboptimal performance in detecting objects under specific environmental conditions and a tendency to favor larger objects over smaller ones, leading to less effective small object detection^22^. Therefore, researchers often refine the model architecture based on the characteristics of their target datasets. Ni et al.^24^ proposed FMR-YOLO, an improved lightweight YOLOv8n model incorporating fast feature extraction, multi-scale fusion, and a receptive-field attention mechanism. It raises $mAP_{50}$ on three industrial defect datasets while substantially cutting parameters and computation. Wang et al.^25^ introduced a lightweight YOLOv5n-based network enhanced with Receptive Field Enhancement, Deformable Convolution, and Bi-level Routing Attention, achieving 82.1% mAP at 60.4 FPS on the NEU-DET dataset for steel surface defect detection. Ma et al.^26^ improved YOLOv5 with a dual-attention module (CBAM) to boost small-object detection accuracy, validated on the WSODD dataset. These studies demonstrate that attention mechanisms offer a straightforward yet effective way to enhance detection performance, as exemplified by the Convolutional Block Attention Module (CBAM) proposed by Woo et al.^27^, which combines channel and spatial attention to focus on salient features while suppressing non-critical information. However, the above models struggle to simultaneously achieve both accurate detection of micro-defects and adequate capture of their salient positional features.

To address the problem of small target miss-detection, numerous researchers have turned their focus to this area of study. In the literature, Lin et al.^28^ qualitatively described small objects as being tiny, occluded, and often set against cluttered backgrounds. Quantitative definitions for small objects mainly follow two approaches^29^. One is based on absolute size; for instance, in the MS COCO dataset, objects with pixel dimensions smaller than $32\times 32$ are defined as small objects. The other is based on relative size; Chen et al.^30^ defined small objects as those whose bounding box area constitutes between 0.08% and 0.58% of the total image area. Compared to other small-object detectors, YOLOv8-based small-object detection primarily faces two challenges^22^: On one hand, there is a cold start problem: During the initial stages of model training, when the model output is disordered, the assignment of positive and negative samples can be ineffective, potentially leading to poor convergence outcomes. On the other hand, the IoU variability problem: small objects exhibit significant Intersection over Union (IoU) fluctuations. Compared to medium and large objects, even a slight offset in the bounding box can cause a substantial change in the IoU value. This sensitivity further exacerbates the challenge of accurately identifying small objects.

### Contribution and organization

In response to the aforementioned diverse challenges and drawing inspiration from recent developments in machine vision, this paper proposes a novel automated inspection method for surface defects on CNC inserts based on machine vision, with experimental validation conducted on square-coated carbide inserts. The main contributions of this study are summarized as follows:

1. An enhanced YOLOv8 model, termed Attention-Augmented Multi-Defects YOLO (A2MD-YOLO): Drawing on the characteristics of this dataset, we introduce a detection model designed to enhance small-target detection by incorporating a CBAM module and a P2 detection head into the YOLOv8 architecture.
2. A complete automated hardware system: For the visual inspection of coated carbide inserts, we integrate technical components including a multi-axis robotic arm to establish a comprehensive hardware system capable of automated defect detection on inserts.
3. Industrial implementation and validation: The proposed algorithm is implemented in an industrial setting and experimentally validated, with evaluations encompassing detection precision, recall, and efficiency.
4. A specialized and publicly accessible dataset: This study constructs a dedicated dataset for insert surface defects and provides a systematic categorization of surface defect types for coated carbide CNC inserts. The dataset is intended to be made publicly available to support further research in related fields.

Section Introduction describes the industrial significance of CNC tool inserts and the critical importance of detecting surface defects on them. It provides a concise review of relevant research in the fields of surface defect detection, small defect detection, and surface defect inspection for metal tools, and subsequently introduces the specific problem addressed in this study along with the proposed methodology. Section Methodology and Dataset elaborates on the overall methodology, detailing the components of the real-time detection system. It comprehensively describes the image acquisition process, the construction of the dataset, and presents a characteristic analysis of the compiled dataset. Section System Model Construction presents the architecture of the A2MD-YOLO and explains the rationale behind the proposed modifications. Section Experimental Setup outlines the experimental setup, including the platform configuration, parameter settings, and the evaluation metrics employed. Section Experiment and Analysis presents comparative experimental results, introduces and discusses ablation studies that validate the effectiveness of the proposed modules, demonstrates visual examples of defects detected in CNC inserts, analyzes the implementation feasibility and real-time performance of the system from an industrial production line perspective, presents a comparison with other hardware systems, and provides an analysis of the system’s uncertainties.Finally, Section Conclusion and Outlook summarizes the work and discusses potential directions for future research.

**Fig. 2:**
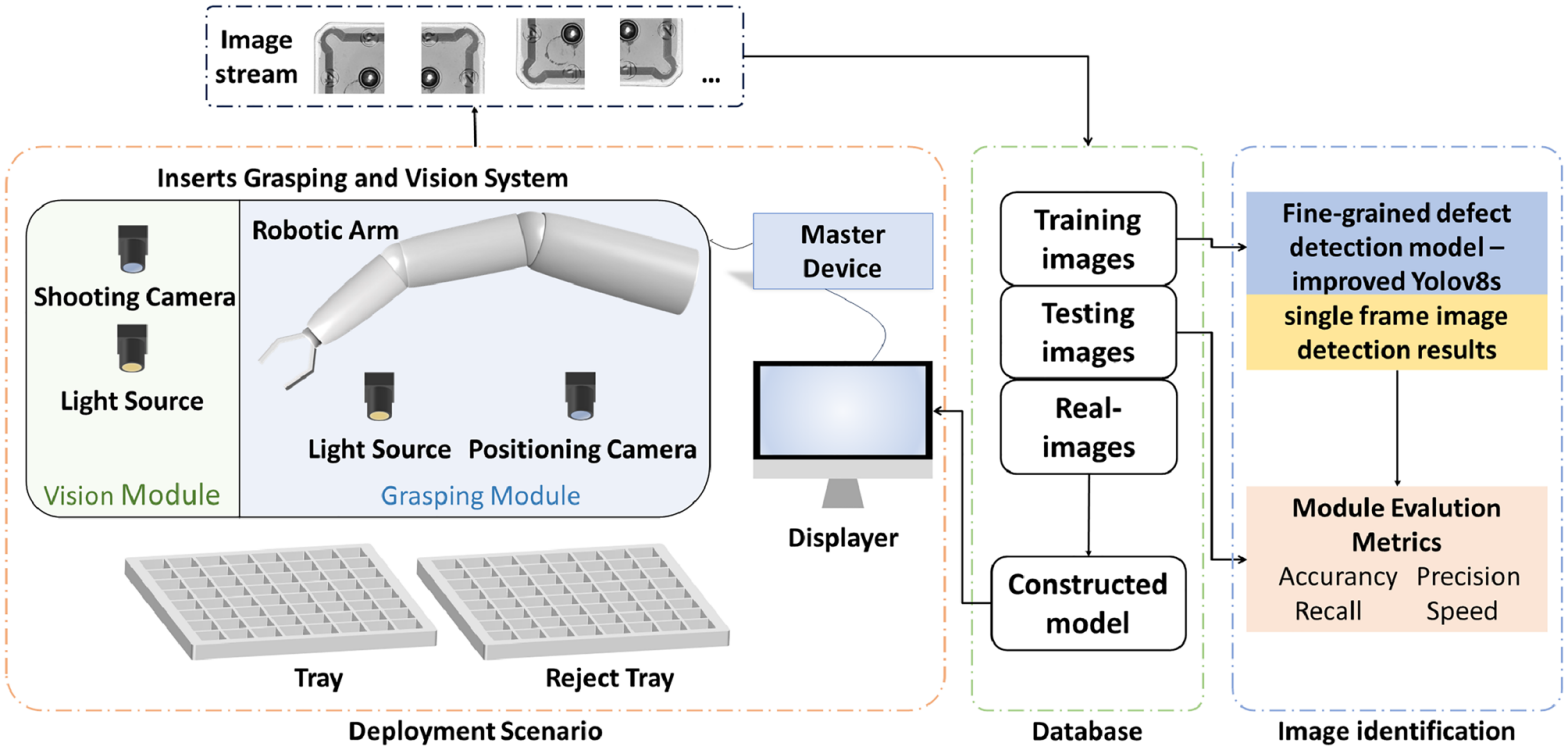
Overview of the proposed methodology, illustrating the workflow from dataset construction and A2MD-YOLO model development to hardware integration and industrial implementation.

## Methodology and dataset

To meet the stringent requirements for both speed and accuracy in industrial defect detection, a system architecture with sophisticated design and optimization is necessary to perform defect detection tasks precisely and efficiently. Figure 2 illustrates the methodology for surface defect detection in CNC tool inserts. The proposed methodology comprises three key components: the development of a hardware system for image acquisition and defect detection, the construction of a comprehensive dataset, and the generation of a dedicated training model. This section will first elaborate on each element of the hardware system to establish a foundation for dataset creation. It will then provide a detailed description of the dataset establishment process, its categorization, and an analysis of its characteristics.

### Image acquisition

**Fig. 3:**
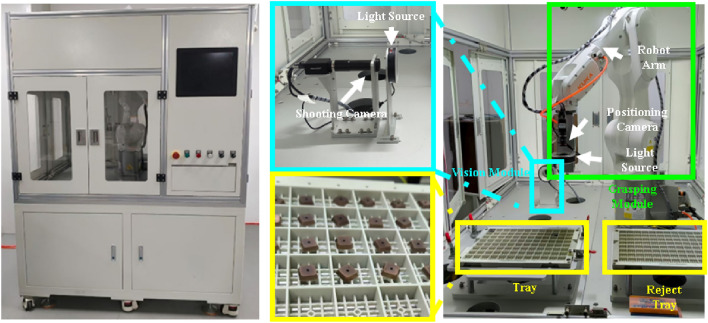
Physical implementation of the proposed automated inspection system, illustrating the overall system appearance and internal integration of key components-including a six-axis robotic arm, a positioning unit, and an imaging unit-as implemented in an industrial setting.

Figure 3 presents a customized hardware experimental platform specifically designed for CNC inserts. Its length and width approximately are $2m \times 2m$.This platform integrates several key components, including an imaging camera, a telecentric lens, a positioning camera, a lens, a high-precision robotic arm (The KUKA KR 6 R900-2 six-axis robotic arm offers a repeatability of ±0.02 mm, providing a fundamental guarantee for precise positioning and imaging of various insert types. With a working radius of 901 mm and support for multiple mounting options, the robotic arm provides the system with excellent flexibility and adaptability, enabling it to accommodate future changes in insert tray size and layout. More importantly, the introduction of the robotic arm also allows the system to adapt to inserts of different materials and shapes, ensuring equipment compatibility.), a lighting source, a material tray, and a defective parts tray. The selection strategy, along with the brands and models of the primary components, is summarized in Table 1.

**Table 1:** Device configuration and selection justification.

Device	Brand	Model	Justification
Imaging camera	HIKROBOT	MV-CH250-90GM/C/N	25MP resolution (5120$\times$5120), pixel size 2.5$\mu$m, enabling detection of small defects.
Telecentric lens	COOLENS	WWK15-110–111	Eliminates perspective error; ensures accurate dimensional measurement for defects at different heights; supports micro-defect imaging.
Positioning camera	HIKROBOT	MV-CU013-A0GM/GC	Provides high-speed, low-latency image acquisition for precise workpiece positioning.
Lens (positioning)	HIKROBOT	MVL-HF1224M-10MP	Compact and cost-effective, suitable for general positioning tasks.
Light source	Panxin	PPX-RI9045-W	Ring LED with bright-field/dark-field composite illumination; $360^{\circ }$ uniform lighting suppresses metal surface reflections and avoids edge blind spots.
Robotic arm	KUKA	KR 6 R900-2	Six-axis robot with ±0.02mm repeatability; enables multi-surface imaging (rake face, flank face, cutting edges) and rapid changeover between insert types.

The light source, as a critical component of machine vision systems, effectively enhances the quality of captured images and significantly improves the contrast between target information and the background. In the detection of micro-defects, the uniformity and brightness of the illumination source directly influence the image clarity of the machine vision system. If the light source is non-uniform or its brightness fails to meet requirements, the detection results can be severely compromised^31^. Therefore, to achieve high-precision, full-field visual inspection of the morphology and defects at the cutting edge of coated carbide square CNC inserts, this study selects a ring LED light source with integrated bright-field and dark-field illumination capabilities as the core lighting solution. This selection is primarily based on the following three considerations:

**Fig. 4:**
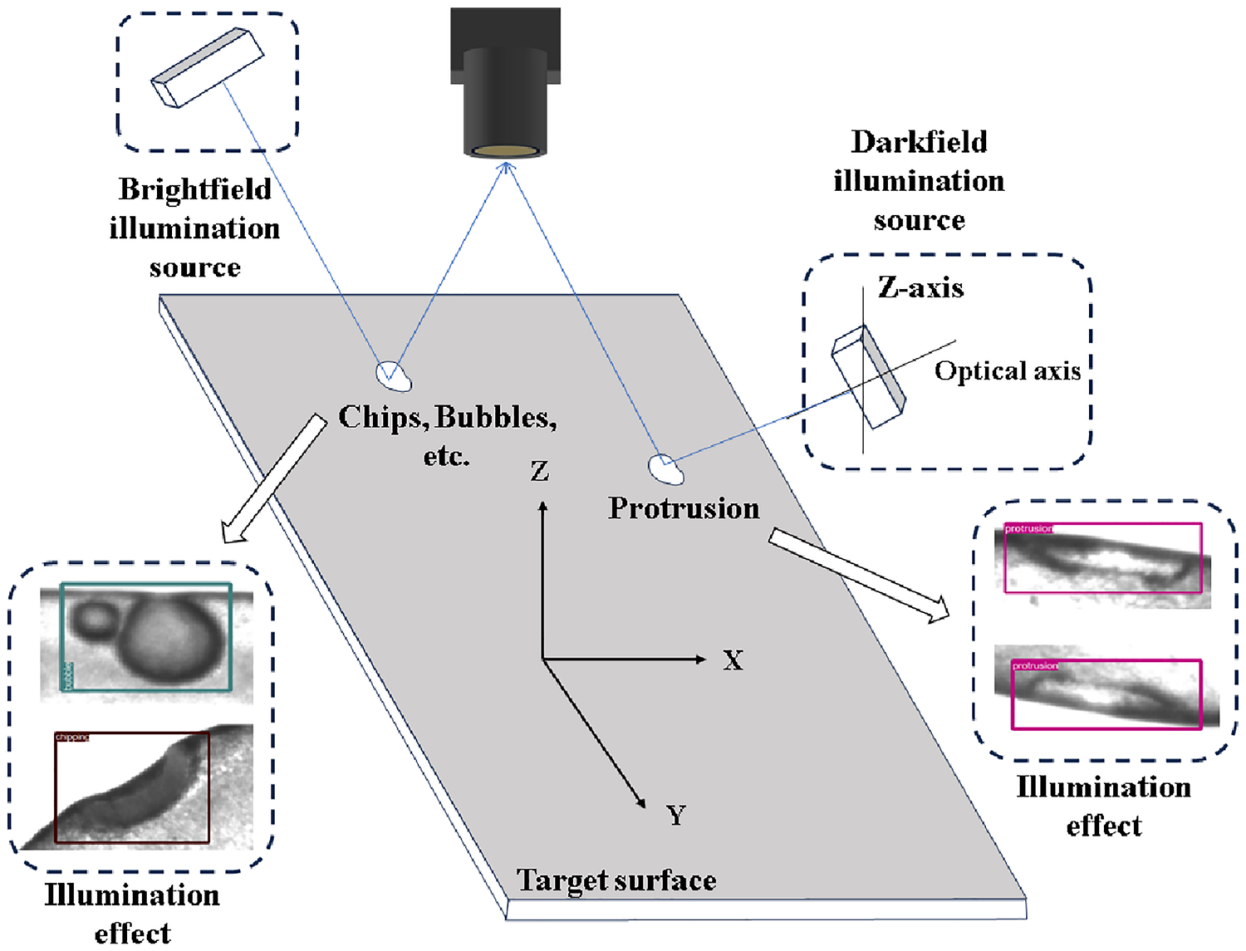
Schematic diagram of the illumination.

**Fig. 5:**
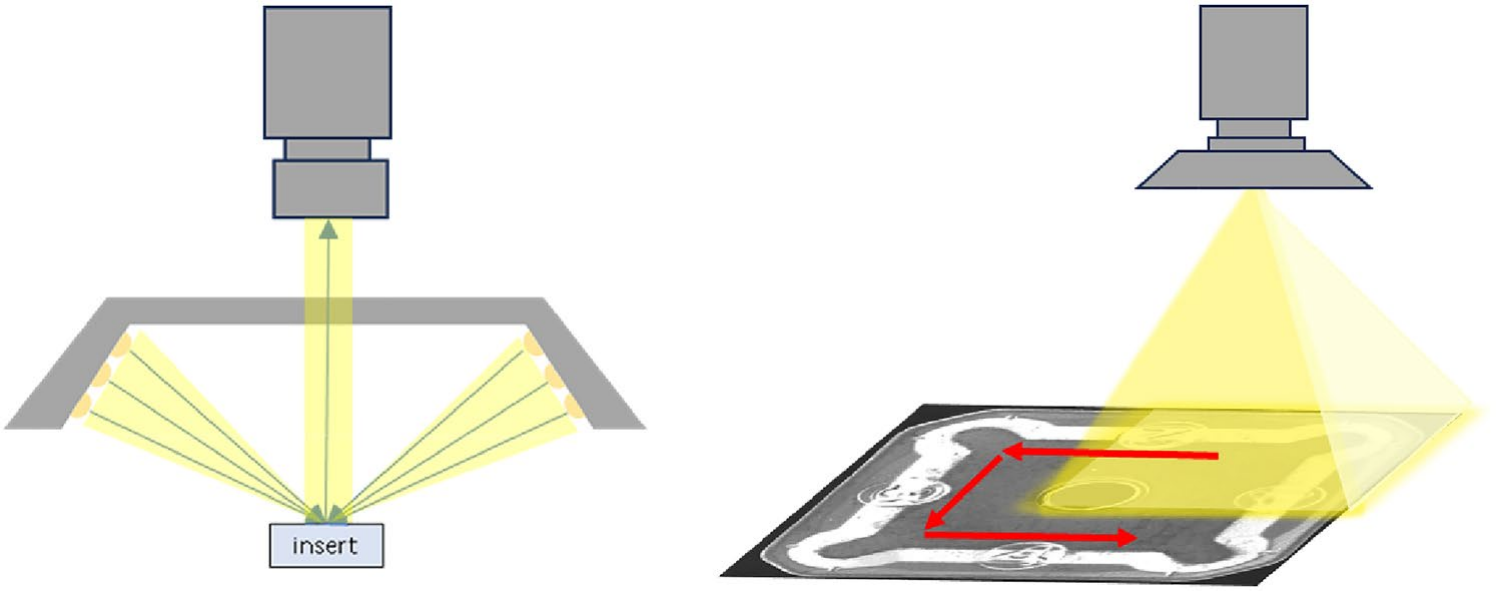
Light source cross-sectional view and camera capture path.

1. At the level of imaging principles, composite lighting fulfills the requirement for the cooperative capture of multiple defect types. The defects to be detected in this experiment exhibit diverse forms: as shown in Fig. 4, on one hand, bright-field illumination (with light incident at small/near-vertical angles) enables defects such as chips and bubbles to appear as dark-contrast features against a bright background due to scattered light loss. On the other hand, dark-field illumination (with light incident at large oblique angles) is highly sensitive to surface undulations, allowing slightly raised defects to appear as bright features against a dark background as scattered light enters the objective lens, thereby avoiding missed detections. A single lighting mode cannot accommodate both scenarios, whereas composite lighting allows the integration of both optical paths in a single acquisition, ensuring simultaneous high-contrast imaging of both “bright” and “dark” defect categories. The light source profile is shown in the left panel of Fig. 5.
2. At the optical control level, this solution effectively suppresses strong reflection interference from the metal surface. The high surface finish of the tool substrate and coating tends to produce specular reflections, leading to localized overexposure and loss of detail. The multi-angle annular light field employed in the composite illumination disperses the reflective glare from a single direction, transforming concentrated, intense reflections into uniform and soft illumination. This significantly enhances image quality, improves the contrast between features and the background, and lays a solid foundation for subsequent processing.
3. From a geometric perspective, the ring light source, with its structural characteristic of omnidirectional angular illumination, is better suited for surface defect detection^32^. The primary subjects of this study are the four straight edges and four corners of the square tool insert. Traditional unidirectional or bilateral light sources are highly prone to uneven illumination due to edge orientation, leading to reduced contrast along certain edges that are unfavorably positioned relative to the light angle. In contrast, the ring light source provides uniform $360^{\circ }$ illumination coverage, ensuring that regardless of edge direction, sufficient and uniform side lighting is obtained. This effectively highlights the morphology of each edge and corner, preventing feature omission caused by lighting blind spots.

The imaging camera is employed to capture high-resolution images of the rake face of the CNC insert. In our experiments, the quadrilateral CNC insert was imaged through four independent shots, with each shot covering an area larger than one-fourth of the complete insert. This approach ensures that no defects along the insert edges are missed after the four imaging cycles, as illustrated in the right panel of Fig. 5.

The positioning camera assists the robotic arm in determining the precise location of the insert. The telecentric lens is primarily used to ensure high-fidelity imaging of surface defects on the insert. The robotic arm possesses six degrees of freedom and offers a repeatability accuracy of ${0.02}\,\hbox {mm}$. The final captured images have a resolution of $5120 \times 5120$ pixels, covering an imaging field of view of ${8.5}\,\hbox {mm}^{2}$, with a pixel size of ${2.5}\,\upmu \hbox {m}$. All equipment employed meets the precision requirements necessary for accurate defect evaluation.

**Fig. 6:**
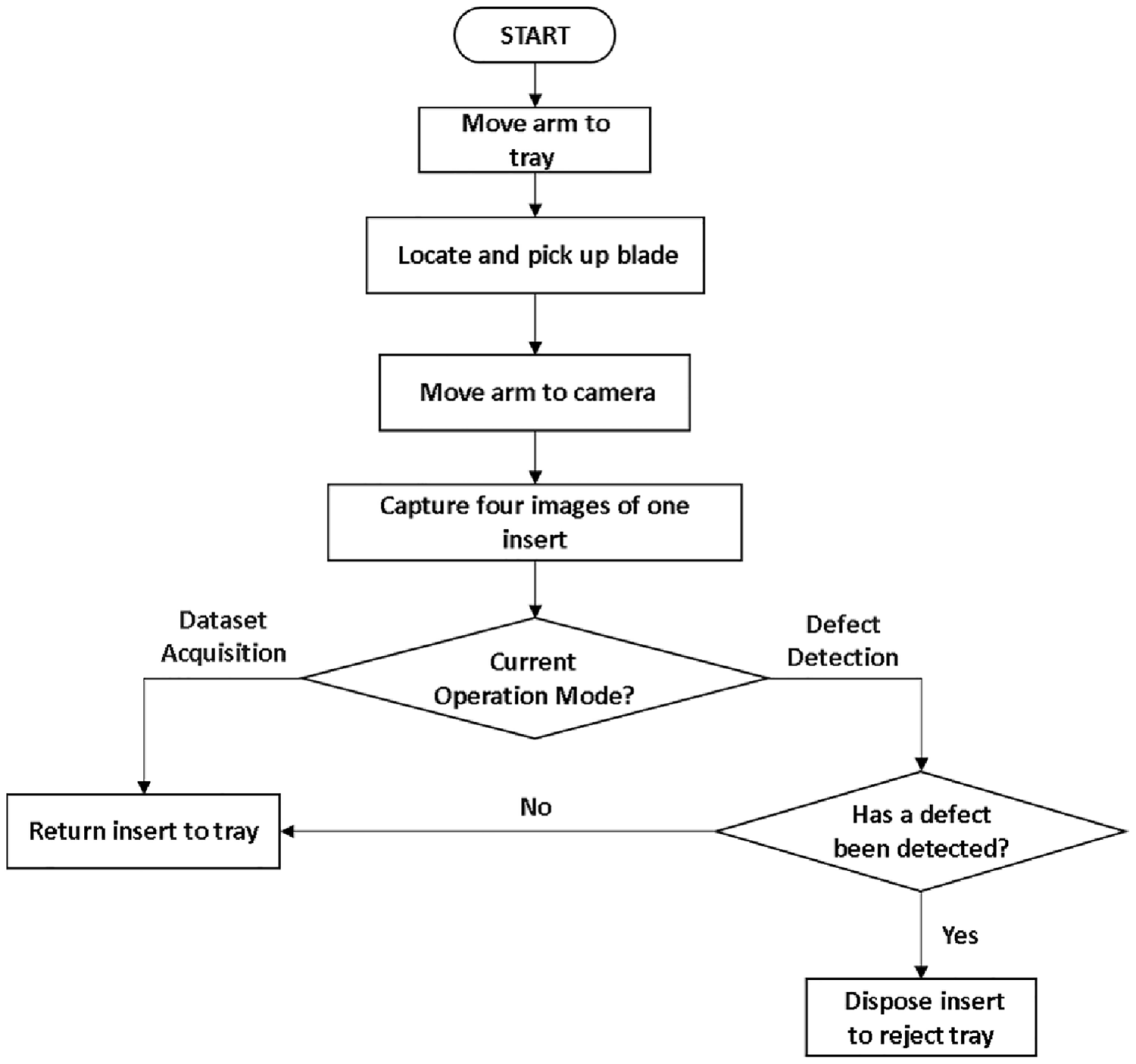
Hardware workflow of the proposed automated inspection system, illustrating the robotic arm operations from gripping the insert from the material tray, positioning, and imaging, followed by a conditional branch: in image acquisition mode, the insert is returned to the material tray; in actual inspection mode, it is sorted into either the defective tray or returned to the material tray based on the detection results.

As shown in Fig. 6, during image acquisition, the insert is first placed into the material tray. The system is then activated, and a positioning camera assists the robotic arm in locating the insert. The robotic arm descends to pick up the insert and moves it to a predefined position in front of the imaging camera. After the imaging camera completes four image captures, the robotic arm returns the insert to its original position, thereby concluding one full cycle of insert data acquisition. During real-time inspection, the robotic arm places the insert either back into the material tray or into a defective material tray based on the inspection results.

**Fig. 7:**
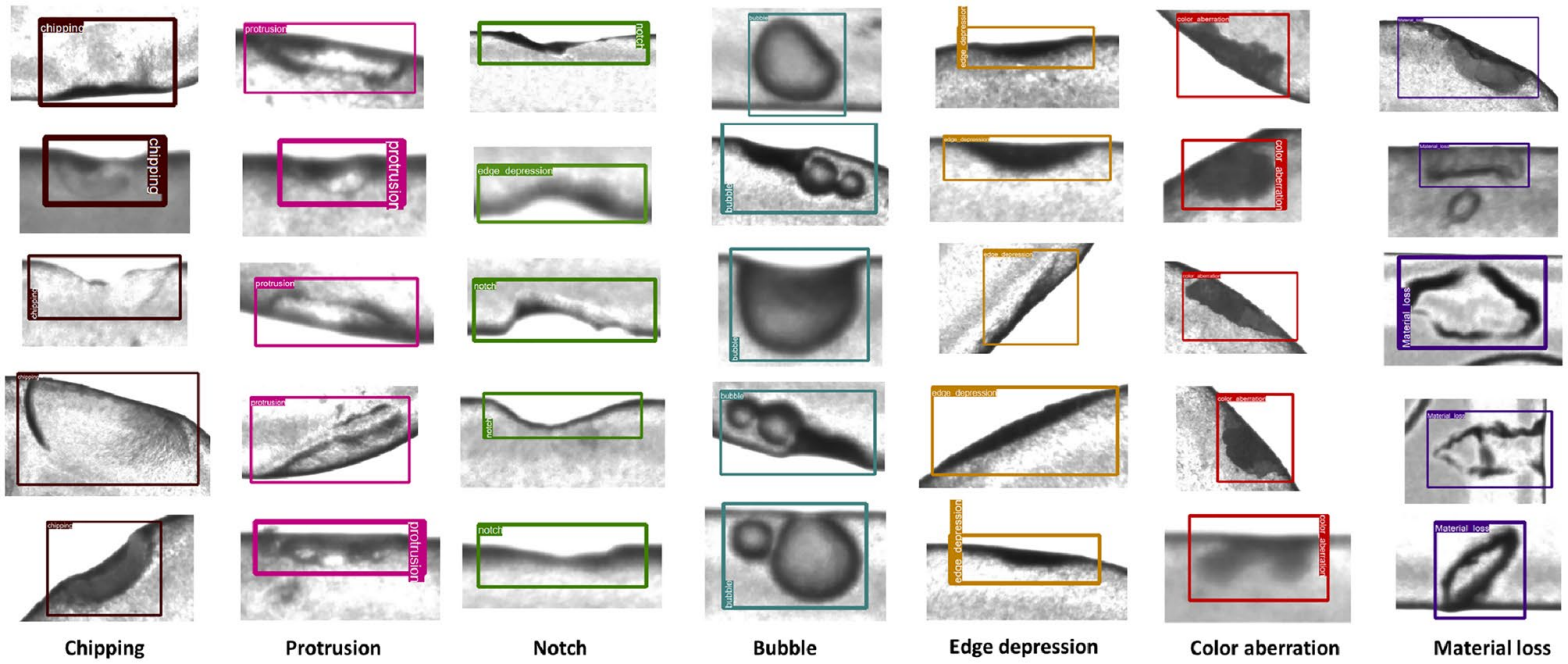
Illustrative examples of the seven annotated defect types.

### Dataset construction

Examples of various defect types are illustrated in the accompanying Fig. 7. This study focuses specifically on defects occurring at or near the cutting edge of CNC tool inserts that affect edge thickness or strength. By capturing images of defective CNC tool inserts, this study constructed a comprehensive dataset comprising 651 images. To enhance the model’s capability in detecting defects, we systematically categorized the surface defects of coated carbide inserts into seven distinct types for the first time and analyzed their morphological characteristics and causes, including: chipping, protrusion, notch, bubble, edge depression, color aberration, and material loss.

Chipping refers to the detachment of metal fragments from the insert edge due to impact, characterized by smooth defect boundaries. Protrusion describes raised material near the cutting edge formed during manufacturing, while the edge itself remains intact. Notch denotes irregularly-shaped indentations along the cutting edge caused by mechanical impact. Bubble defects, formed during manufacturing, appear as circular or overlapping circular features with low edge brightness and higher basal luminance. Color aberration results from coating delamination or similar issues, exhibiting distinct curved demarcation lines between color shades without significant internal brightness variation. Material loss indicates irregularly-contoured absence of metal at or near the cutting edge caused by manufacturing imperfections. Edge depression refers to manufacturing-induced edge concavities with regular contours and low luminance. Figure 7 illustrates representative sample images of the seven defect categories.

All images were annotated using X-AnyLabeling (version 2.5.3)^33^ with rectangular bounding boxes.The annotation format follows the COCO format, which includes bounding box coordinates and category labels. During annotation, we adhere to the following rules: (a) Bounding boxes follow the principle of the minimum enclosing rectangle to ensure tight coverage of the defect region. (b) For two or more adjacent defects of the same type, if the distance between them is less than half the size of the smallest defect among them, they are annotated as a single defect rather than separately. This rule is intended to better reflect the holistic assessment of defect regions in practical industrial inspection. (c) This study focuses on edge defects of the insert; therefore, for non-edge defects that may affect the edge thickness of the insert (e.g., bubbles near the edge), the relevant edge area is also included in the annotation to fully capture the impact of the defect on the edge structure. To ensure annotation quality, each image was independently annotated by two authors. In cases where annotations were inconsistent, a third author adjudicated to reach a final consensus.

The dataset was randomly partitioned into a training set (522 images) and a validation set (129 images) at a ratio of 4:1. The validation set was employed for both model selection and final performance evaluation, with no separate test set being additionally partitioned. To mitigate the impact of class imbalance on model training, limited data augmentation was applied to the “protrusion” category, which initially contained the fewest samples. Specifically, the original four training samples were expanded to twenty samples through random rotation and horizontal flipping operations. All augmentation operations were applied exclusively to the training set, while the validation set remained in its original acquisition state without any augmentation to ensure objective and authentic performance assessment. No excessive balancing augmentation was applied to the other categories, thereby preserving the real-world distribution characteristics of the dataset. The final numbers of images for each defect category in both the training and validation sets are detailed in Table 2.

**Table 2:** Sample distribution per category.

Defect category	Training instances	Validationinstances
Chipping	372	94
Protrusion	20	1
Notch	205	45
Bubble	74	12
Edge depression	176	28
Color aberration	47	14
Material loss	112	26
**Total**	1006	220

### Characteristic of dataset

Upon completion of the dataset construction, we performed a comprehensive analysis of its characteristics to inform potential model architecture refinements for enhanced detection performance. As evidenced in the accompanying Fig. 7, substantial intra-class^34^ variation exists where defects within the same category exhibit significant morphological diversity. For instance, specimens under the “material loss“ category demonstrate considerable differences in both size and geometrical configuration. Conversely, notable inter-class similarity is observed between different defect types, as exemplified by the visual resemblance between “notch” and “edge depression” categories. This inherent cross-category resemblance coupled with within-category heterogeneity presents considerable challenges for accurate defect classification. Furthermore, the dataset exhibits pronounced scale variation across defect types. Within the “chipping” category, for example, larger instances measure approximately $1000 \times 500$ pixels while smaller manifestations are merely $400 \times 200$ pixels. The “notch” defects generally display smaller dimensions, with most instances measuring around $300 \times 100$ pixels. Such significant scale disparity complicates the development of robust defect detection algorithms.

## System model construction

**Fig. 8:**
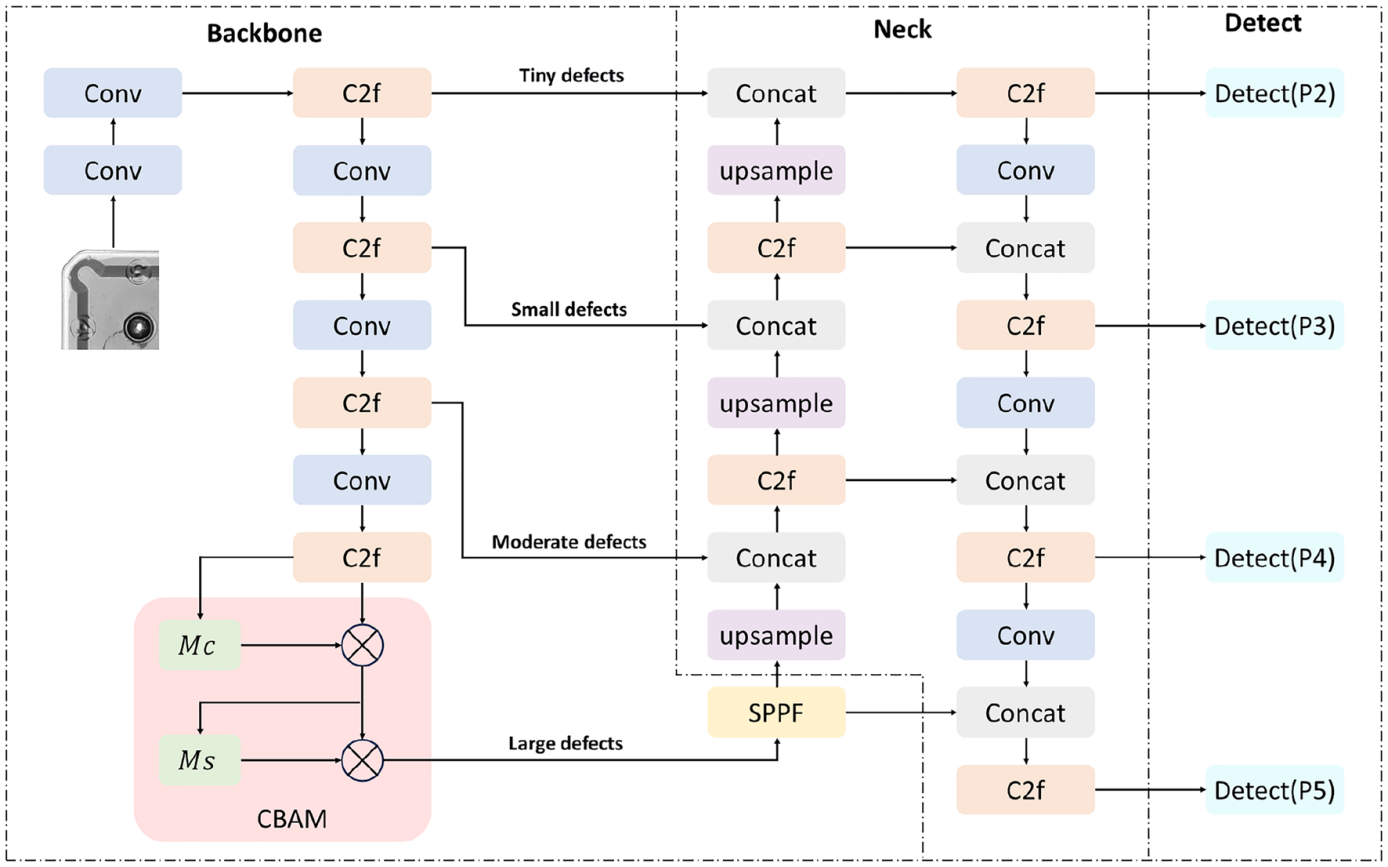
Network structure of A2MD-YOLO.

To address the aforementioned issues, this paper proposes the A2MD-YOLO model. Based on YOLOv8, the model incorporates targeted improvements, with its overall architecture illustrated in Fig. 8. Specifically, the CBAM module is introduced into the Backbone, and a dedicated P2 detection head for small object detection is added to the Detect module.

### P2 detection head

To address the issue of missed detections caused by smaller defects in the dataset, this study introduces an additional P2 detection head into the model. The standard YOLOv8 model typically utilizes P3, P4, and P5 features for object detection, where higher-level layers possess larger receptive fields. These higher-level features with larger receptive fields tend to prioritize global information, often at the expense of local details crucial for small object detection. The YOLOv8-P2 is a variant of YOLOv8 specifically optimized for small object detection. To enhance its capability in detecting small targets, YOLOv8-P2 incorporates a detection head at the P2 layer, which undergoes only two convolutional operations and retains a larger feature size. The introduction of this detection head enables shallower features to participate in the detection process. These shallow features contain more detailed information and offer higher resolution for capturing the edges and shapes of small objects.

Furthermore, in the architecture of YOLOv8-P2, the original Neck section is augmented with one upsampling module, three C2f modules, two convolutional modules, and three concatenation modules. This design integrates the features from the P2 layer with the existing P3, P4, and P5 features for detection, thereby enhancing the saliency of the features of the detected objects.

### CBAM

In this study, we introduce CBAM to guide the model in more effectively learning the unique distribution patterns of insert defects. Given that the defects in our dataset are stably and consistently distributed along the insert edges-exhibiting strong spatial regularity–and that intra-class defects exhibit significant feature variance while inter-class defects share similarities, CBAM’s synergistic channel-spatial attention mechanism is particularly suitable. The channel attention directs the model to focus on feature maps most relevant to defects (e.g., edges, textures), whereas the spatial attention enables it to actively pinpoint high-frequency defect regions, specifically the spatial locations along the insert edge. Through this dual optimization of “what” and “where” is important, the model achieves precise enhancement of defect features and effective suppression of background noise.

Specifically, the CBAM module is embedded at the end of the backbone, preceding the SPPF layer. This placement strategy is motivated by the following rationale: after multiple layers of convolution and downsampling in the backbone, the intermediate feature maps at this stage possess a large receptive field and contain rich global contextual information. Introducing CBAM at this point allows for the recalibration of feature responses based on global perception, thereby significantly improving the model’s ability to detect medium and large defects, as accurate identification of such defects often relies on a comprehensive understanding of their overall spatial structure and context. This design complements the P2 detection head, which is specifically introduced for detecting minute defects, together forming a robust detection system capable of handling defects across various scales.

A schematic diagram of the model’s two sub-modules, the Channel Attention Module and the Spatial Attention Module, the complete attention process can be described as follows^27^:

1 $$\begin{aligned} F'=M_{c}(F) \circ F \end{aligned}$$

2 $$\begin{aligned} F''=M_{s}(F') \circ F' \end{aligned}$$

Among them, for the input intermediate feature map $F \in \mathbb {R}^{C \times H \times W}$, CBAM will sequentially generate two attention maps: a 1*D* channel attention map $M_{c}$ and a 2*D* spatial attention map $M_{s}$.$\circ$ denotes element-wise multiplication. During multiplication, attention values are appropriately replicated: channel attention values are broadcast along the spatial dimensions, and vice versa. $F''$ is the final output.

## Experimental setup

### Experimental details

The hardware and software configurations of the experimental platform are detailed in Table 3.

**Table 3:** Configurations of the experimental platform and training hyperparameters.

**Experimental setup**	
Hardware Configuration	
CPU	12th Gen Intel(R) Core(TM) i9-12950HX
GPU	NVIDIA RTX A4500 Laptop (16GB VRAM)
RAM	64 GB
Training Hyperparameters	
Input image size	640 $\times$ 640
Batch size	16
Epochs	1000
Optimizer	SGD
IoU threshold	0.5
Conf	0.2
Software Environment	
Python	3.9.19
PyTorch	2.2.2
CUDA	11.8
Ultralytics	8.3.100

### Performance evaluation metrics

To evaluate model performance, this study employs five key metrics: Precision, Recall, $mAP_{50}$, $mAP_{50-95}$, and the $F_{\beta }$-Score. Their definitions are as follows, where FP, TP, FN, and TN denote False Positives, True Positives, False Negatives, and True Negatives, respectively.

1. Precision: Precision reflects the probability that a target detected by the model is indeed a true defect. Its calculation formula is as follows:
  3 $$\begin{aligned} \textrm{precision}=\frac{\textrm{TP}}{\mathrm {TP+FP}} \end{aligned}$$
2. Recall: Recall reflects the model’s completeness in identifying defects, i.e., its ability to detect all actual defects. The calculation formula is as follows:
  4 $$\begin{aligned} \textrm{Recall}=\frac{\textrm{TP}}{\mathrm {TP + FN}} \end{aligned}$$
3. $mAP_{50-95}$: Average Precision (AP) comprehensively reflects the overall performance of a model by calculating the precision at different recall rates on the precision-recall curve. $mAP_{50-95}$ represent the *mAP* values at IoU (Intersection over Union) thresholds of $50-95\%$. $mAP_{50-95}$ takes both precision and recall into consideration, providing a more comprehensive evaluation of model performance. The calculation formula is as follows:
  5 $$\begin{aligned} \textrm{AP} = \int _{0}^{1} \mathrm {precision(recall) d(recall)} \end{aligned}$$
  6 $$\begin{aligned} \mathrm {mAP_{50-95}}=\mathrm {\frac{1}{N}\sum _{i=1}^{N}AP_{i}^{[0.5,0.95]}} \end{aligned}$$
  7 $$\begin{aligned} \textrm{IoU} = \frac{|\textrm{Prediction} \cap \textrm{Truth}|}{|\textrm{Prediction} \cup \textrm{Truth}|} \end{aligned}$$
4. $F_{\beta }-Score$:Precision and Recall are typically conflicting metrics; an increase in the decision threshold tends to elevate Precision at the expense of Recall, whereas a decrease in the threshold improves Recall but compromises Precision. To holistically evaluate both, the $F_{\beta }-Score$ is adopted, which is defined as follows:
  8 $$\begin{aligned} \mathrm {F_\beta } = \mathrm {\frac{(1+\beta ^2)\times (Precision\times Recall)}{\beta ^2 \times Precision + Recall}} \end{aligned}$$
  Here, $\beta$ is a parameter that balances the importance between Precision and Recall. When $\beta =1$, Precision and Recall are considered equally important. A value of $\beta <1$ assigns greater weight to Precision than to Recall, while $\beta>1$ indicates that Recall is prioritized over Precision. In the context of industrial defect detection, the primary objective is often to identify the presence of defects rather than to classify them into specific categories precisely. Furthermore, since missed detections (false negatives) can lead to severe quality risks, while a certain proportion of false alarms (false positives) can be rectified through subsequent manual review, this scenario imposes a stricter requirement for a low missed-detection rate. Consequently, in this work, we set $\beta$ to 3. The formula for the $\text {F}_{3}-\text {Score}$ is thus given by:
  9 $$\begin{aligned} \mathrm {F_3} = \mathrm {\frac{10\times (Precision\times Recall)}{9 \times Precision + Recall}} \end{aligned}$$

## Experiment and analysis

### Comparative experiment

Given its maturity and stability in industrial applications, YOLOv8 is selected as the baseline model in this study. In light of the continuous rapid evolution of the YOLO series, which has produced subsequent versions such as YOLOv11, YOLOv12, and YOLO26(released in 2025), a comparative experiment among different versions is first conducted. In this experiment, the performance of YOLOv8, YOLOv11, YOLOv12, YOLO26, and the proposed A2MD-YOLO model is evaluated on the same test set, with the results summarized in Table 4. The experimental results indicate that YOLOv8 outperforms YOLOv11, YOLOv12, and YOLO26 in terms of both precision and $\text {mAP}_{50-95}$, while A2MD-YOLO achieves higher recall, $\text {F}_{3}-\text {Score}$, and $\text {mAP}_{50-95}$ compared to all the baseline models.

**Table 4:** Performance of different Yolo models.

Module	Precision	Recall	$F_3$	$mAP_{50-95}$
YOLOv8	**0.791**	0.595	0.610	0.529
YOLOv11	0.754	0.640	0.650	0.486
YOLOv12	0.774	0.55	0.566	0.459
YOLO26	0.654	0.677	0.675	0.518
**A2MD-YOLO**	0.661	**0.733**	**0.725**	**0.571**

### Ablation experiment

In the second phase, an ablation study was carried out based on YOLOv8. Three enhanced model configurations were developed: a model incorporating a CBAM module prior to the SPPF module (YOLOv8-CBAM), a model augmented with a P2 detection head (YOLOv8-P2), and our proposed model integrating both the CBAM module and the P2 detection head (A2MD-YOLO). All models were trained under the same conditions and evaluated on an independent test set. Detailed comparative results are provided in Table 5.

**Table 5:** Performance of ablation experiment on Yolov8.

Module	Precision	Recall	$F_3$	$mAP_{50-95}$
YOLOv8	**0.791**	0.595	0.610	0.529
YOLOv8-CBAM	0.716	0.705	0.706	0.55
YOLOv8-P2	0.715	0.7	0.702	0.559
**A2MD-YOLO**	0.661	**0.733**	**0.725**	**0.571**

The ablation study results indicated a consistent performance trade-off pattern across the three enhanced models relative to the baseline YOLOv8. While a moderate decrease in precision was observed, noticeable improvements were achieved in recall, $\text {mAP}_{50-95}$, and the $\text {F}_{3}-\text {Score}$. This trend suggests the potential effectiveness of the introduced modules. Specifically, the proposed A2MD-YOLO model demonstrated a distinct trade-off profile: precision decreased from 0.791 to 0.661, while recall increased substantially from 0.595 to 0.733. Correspondingly, the $\text {F}_{3}-\text {Score}$ improved from 0.610 to 0.725, and $\text {mAP}_{50-95}$ rose from 0.529 to 0.571.

These findings imply that the proposed model can maintain considerable detection reliability while substantially improving defect coverage, thereby achieving a more balanced overall performance. This characteristic appears to align well with the target application scenario, as in industrial quality inspection, the potential cost of overlooking a defect often outweighs that of a false alarm.

**Table 6:** Correct detection and miss rate of various models.

Model	Correct detection rate	Miss rate
YOLOv8	63.6	21.4
YOLOv8-P2	71.4	12.3
YOLOv8-CBAM	67.7	18.6
A2MD-YOLO	73.2	11.8

Table 6 presents the detection accuracy and miss rate of the four models on the validation set, which contains 220 defects. As shown in the Table 6, both the YOLOv8-P2 and YOLOv8-CBAM models demonstrate improved detection accuracy compared to the baseline YOLOv8 model, along with a reduction in miss rate. Notably, the A2MD model exhibits the most substantial improvement: its detection accuracy increases from 63.6% to 73.2%, while the miss rate decreases from 21.4% to 11.8%. This comparison provides strong evidence for the effectiveness of the introduced module, indicating a meaningful alleviation of the miss detection issue.

In summary, although the absolute improvement in $\text {mAP}_{50-95}$-the most stringent metric in object detection-may appear modest, the reduction in miss rate, the substantial gains on challenging small defects, the improvement in $\text {F}_{3}-\text {Score}$, together with the rigorous ablation validation, collectively demonstrate the significant effectiveness of the proposed method for industrial surface defect detection.

**Fig. 9:**
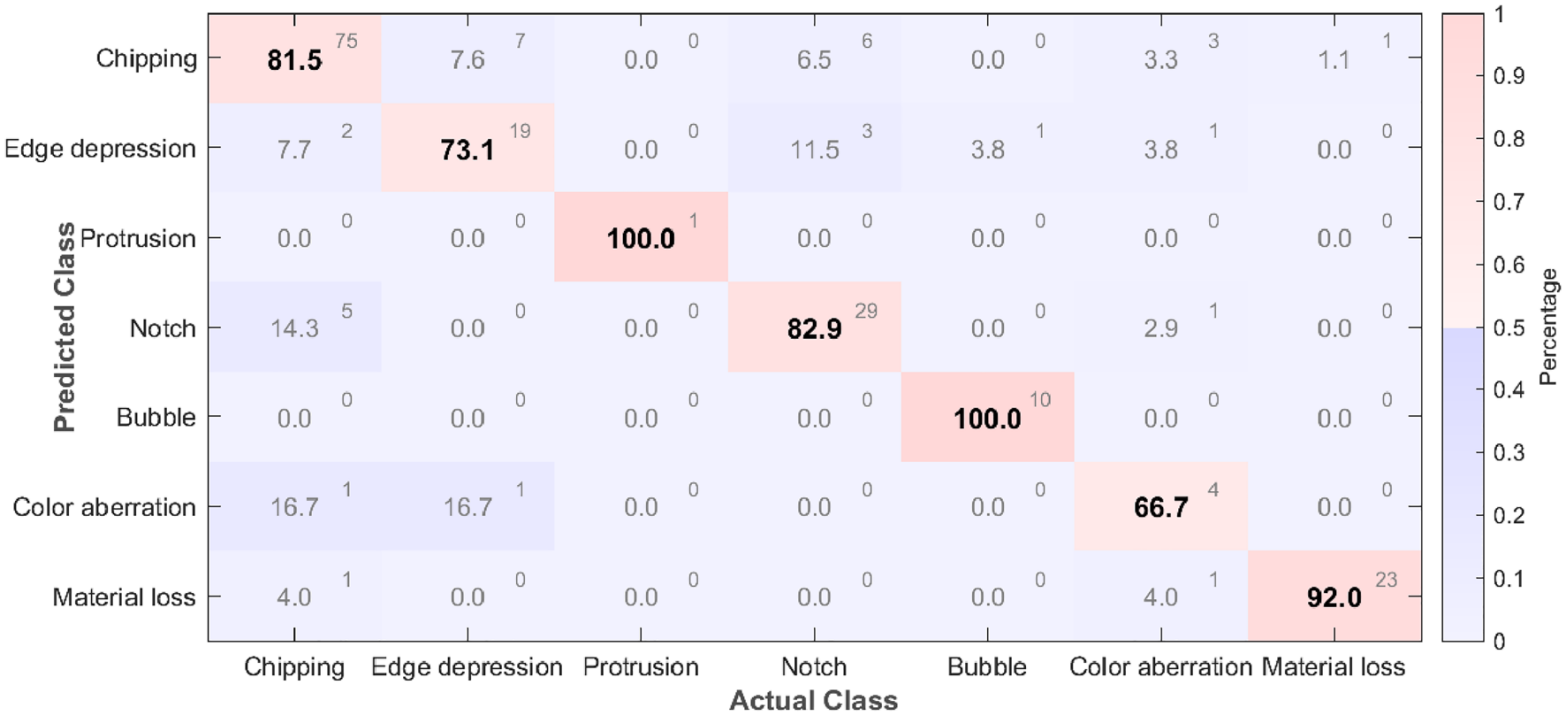
Confusion matrix of the proposed A2MD-YOLO model for the seven defect categories on coated carbide CNC inserts.

**Fig. 10:**
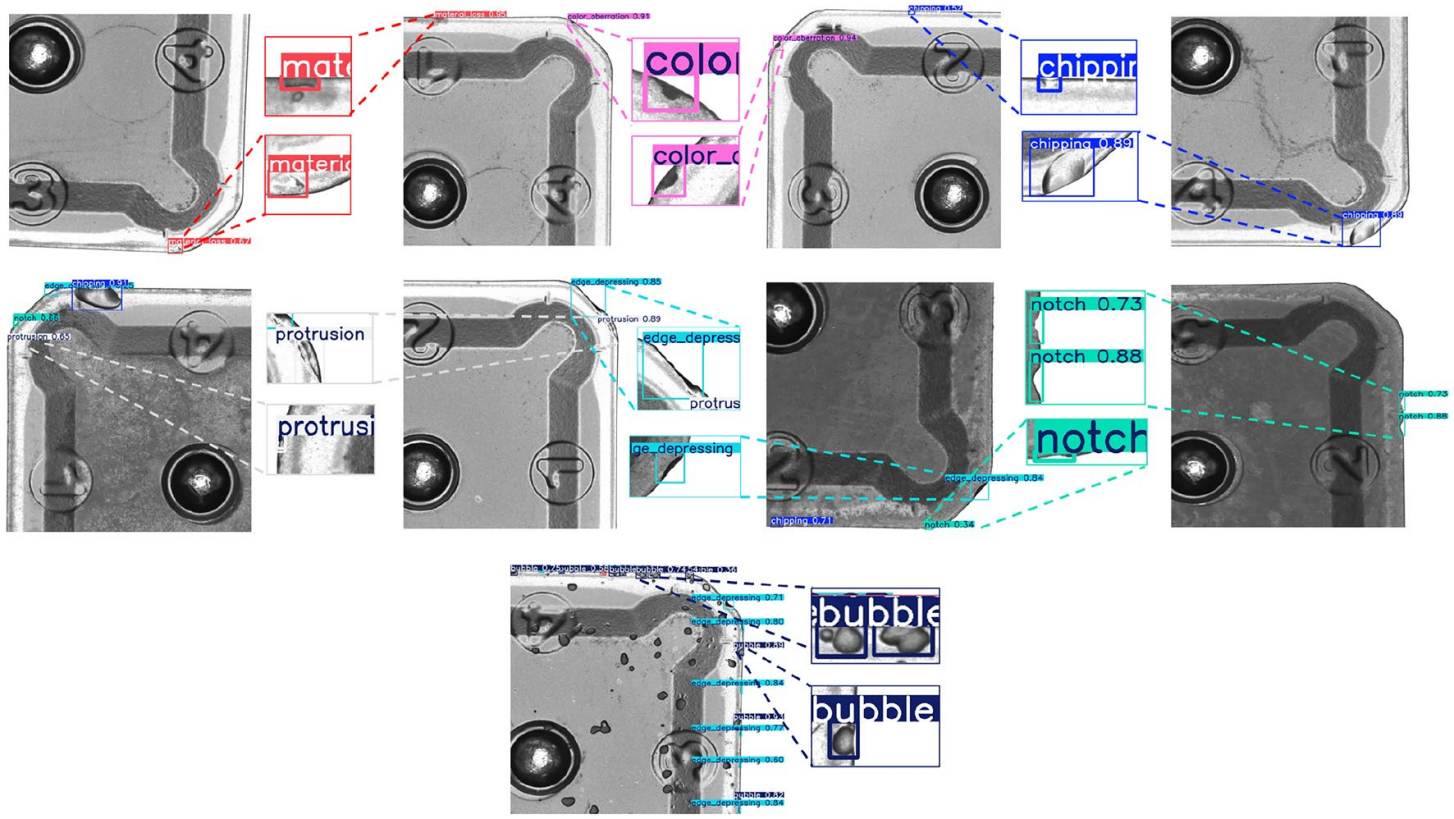
Detection results (Row 1: Material loss, Color aberration, Chipping; Row 2: Protrusion, Edge depression, Notch; Row 3: Bubble).

Figure 9 presents the normalized confusion matrix of the A2MD-YOLO model’s predictions, where the actual number of defects is annotated in the upper-right corner of each cell. The confusion matrix is a tabular tool used to evaluate the performance of classification models, providing an intuitive representation of the model’s discriminative accuracy across different categories. In this matrix, rows correspond to the true labels, while columns represent the predicted labels. To mitigate bias caused by imbalanced sample sizes, the matrix is normalized, thereby offering a more equitable visualization of the model’s independent performance per category. After normalization, each cell value is converted into the percentage of samples belonging to that category, with each row summing to 1.

From the Fig. 9, it can be observed that for the two defect types with distinctive features-protrusion and bubble-the model achieves perfect detection on the test set. In contrast, a small number of misclassifications occur among the remaining defect categories that share similar characteristics. In the practical application of CNC insert defect detection, such misclassified defects can be corrected through manual re-inspection, and the associated cost is far lower than the quality risk caused by missed defects. Therefore, this performance aligns well with real-world engineering requirements.

### Industrial implementation

#### Industrial inspection results

We load the A2MD-YOLO model into the hardware system illustrated in Fig. 3 for validation, Fig. 10 depicts the detection results for each type of defect. It can be observed from the figure that the model successfully detects defects of varying sizes. A representative Chipping defect (first row, fourth column) measures $476 \times 538$ pix ($\approx 1.2 \times 1.4 \,\hbox {mm}$), while a much smaller Notch (second row, third column) is only $126 \times 40$ pix ($\approx 0.3 \times 0.1 \, \hbox {mm}$). Simultaneously, the results in the third row notably show the model’s specific sensitivity to defects located on the cutting edge, disregarding other non-relevant areas.

#### Analysis and comparison of industrial inspection results

**Fig. 11:**
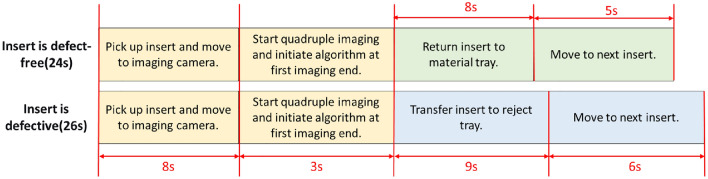
Breakdown of per-insert inspection time for non-defective and defective inserts, illustrating the time distribution across four operational stages.

In practical applications, the proposed system achieves an average processing time of approximately 25 seconds per CNC tool insert. Figure 11 illustrates the per-insert inspection timeline, where minor fluctuations in actual duration can arise from factors such as insert positioning within the material tray and variations in detection outcomes. Based on this average time, the inspection of a full tray ($12 \times 8$ inserts) can be completed in about 40 minutes.Based on official disclosure from the Tianyuan District Government, a medium-sized CNC insert manufacturer has an annual production capacity of 10 million inserts, corresponding to approximately 5,000 inserts per hour (equivalent to about 0.72 seconds per insert) assuming an eight-hour workday and 250 working days per year^35^, while manual visual inspection takes approximately 2 seconds per insert. It should be noted that the system is not intended to replace real-time online inspection at the production cycle level, but rather to achieve fully automated end-to-end processing-from the workpiece leaving the machine tool, through visual inspection and automatic sorting, to sealing and packaging-thereby replacing manual labor in the quality inspection stage while supporting 24-hour continuous operation. Within this context, the current inspection time already provides the necessary conditions to support the operation of an automated production line. Furthermore, the inspection time remains amenable to further optimization. In future work, we aim to shorten the inspection cycle and enhance efficiency by optimizing the motion path of the robotic arm and reducing the travel distance.

We compared this study with a conventional optical microscopy system (e.g., the Keyence VHX-X1 series^36^) and the most closely related existing work (Zeng et al., 2024^7^), and the comparison results are presented in Table 7.

Compared with conventional optical microscopy and the most relevant existing study by Zeng et al.^7^, the proposed system demonstrates several notable advantages. First, unlike optical microscopy, which mainly relies on manual focusing and visual observation and is therefore highly operator-dependent, our system achieves a fully automated inspection workflow integrating gripping, positioning, imaging, and sorting by means of a six-axis robotic arm. In contrast to Zeng’s conveyor-belt-based solution, which is limited to fixed-angle imaging and likely still requires manual loading and unloading, the proposed system enables multi-surface inspection of coated carbide CNC inserts, covering the rake face, flank face, and cutting edges. Second, from the perspective of data resources, our work establishes the first publicly available defect dataset for coated carbide CNC inserts, including seven defect categories together with detailed characteristic analysis, whereas Zeng’s study considered only four defect categories and did not provide a public dataset. Third, in terms of intelligent inspection capability, our method adopts the proposed A2MD-YOLO model, which is more specifically tailored to the present inspection task than the closed-source manufacturer solutions used in optical microscopy systems and the improved YOLOv5 adopted in previous work. Overall, while optical microscopy remains accurate but expensive and heavily manual, and Zeng’s method offers a relatively low-cost semi-automated solution, our approach provides a better balance among automation, inspection completeness, dataset contribution, and practical deployment potential for coated carbide insert defect inspection.

**Table 7:** Comparison between the proposed system, optical microscopy, and the most relevant existing study.

Aspect	Optical Microscopy^36^	Zeng’s^7^	Ours
Insert Material	Any	Cubic boron nitride	Coated carbide
Dataset for Defect	N/A	Four defect categories; not publicly available	First publicly available dataset for coated carbide CNC inserts with seven defect categories; detailed characteristic analysis
Machine Vision Model	Manufacturer Closed-Source	Improved YOLOv5	A2MD-YOLO
Operation Level	Manual operation; manual focusing and observation; operator dependent	Conveyor-belt-based system; fixed-angle imaging; likely manual loading/unloading; single-surface inspection	Six-axis robotic arm; fully automated ready for gripping, positioning, imaging, and sorting; multi-surface inspection (rake face, flank face, cutting edges)
Industrial Automation-Ready Level	Low	Medium	High
Cost	High	Low	Medium

#### Current status and future prospects of industrial deployment

The system is currently implemented as an independent inspection unit. In operation, the operator is only required to place the material tray containing the inserts to be inspected at the designated station, after which the system automatically performs the entire workflow–from gripping and imaging to detection and sorting–while the removal of the tray is conducted manually upon completion. This deployment mode entails no structural modifications to existing production lines and offers straightforward installation and commissioning. It is suitable for offline quality inspection, batch sampling, and auxiliary inspection at the end of production lines. General production workers can be trained in a short period to handle tasks such as tray placement, equipment start-up and shutdown, and basic maintenance, and the system is capable of 24-hour continuous operation without requiring sustained manual intervention.

From a cost-effectiveness perspective, the core advantage of this system lies in replacing manual inspection with automation, thereby enhancing the stability and consistency of inspection results and eliminating the variability associated with subjective judgment in manual operations. On one hand, the one-time equipment investment and long-term maintenance costs are substantially lower than the labor costs associated with multiple inspectors under equivalent production capacity. On the other hand, the system can operate continuously 24/7, effectively avoiding quality complaints and return losses caused by missed detections due to inspector fatigue, while also reducing efficiency fluctuations and hidden management costs associated with personnel turnover and training cycles, thereby further improving overall cost-effectiveness. Although the initial investment is relatively high, the system demonstrates considerable economic value in terms of both labor substitution and quality improvement from a long-term operational perspective.

In terms of practical feasibility, the system exhibits favorable conditions for deployment in industrial environments. The core hardware components are industrial-grade, featuring vibration resistance, anti-interference capability, and long-term stable operation, making them well-suited to typical factory conditions. The system achieves end-to-end automation from gripping and imaging to sorting, adopts a modular design with a small footprint, and has minimal impact on existing production line layouts, facilitating flexible deployment. Moreover, the system has undergone continuous testing in real production environments, demonstrating stable operation and consistent detection performance, thereby validating its industrial reliability.

In the future, the system can be further integrated into automated production lines to achieve fully unmanned inspection. Specifically, deeper integration with production lines can be realized in two ways: first, by interfacing with upstream and downstream processes via conveyors or automatic loading and unloading devices, enabling inserts to enter the inspection unit automatically within the production cycle; second, through signal interaction to enable communication with production line control systems, allowing inspection results to be fed back in real time to production scheduling systems for sorting control or process adjustment. At that stage, the system will evolve from its current semi-automated mode into a fully automated inspection station synchronized with the production cycle, thereby further enhancing overall production efficiency and intelligent capabilities.

### Detection uncertainty analysis

**Fig. 12:**
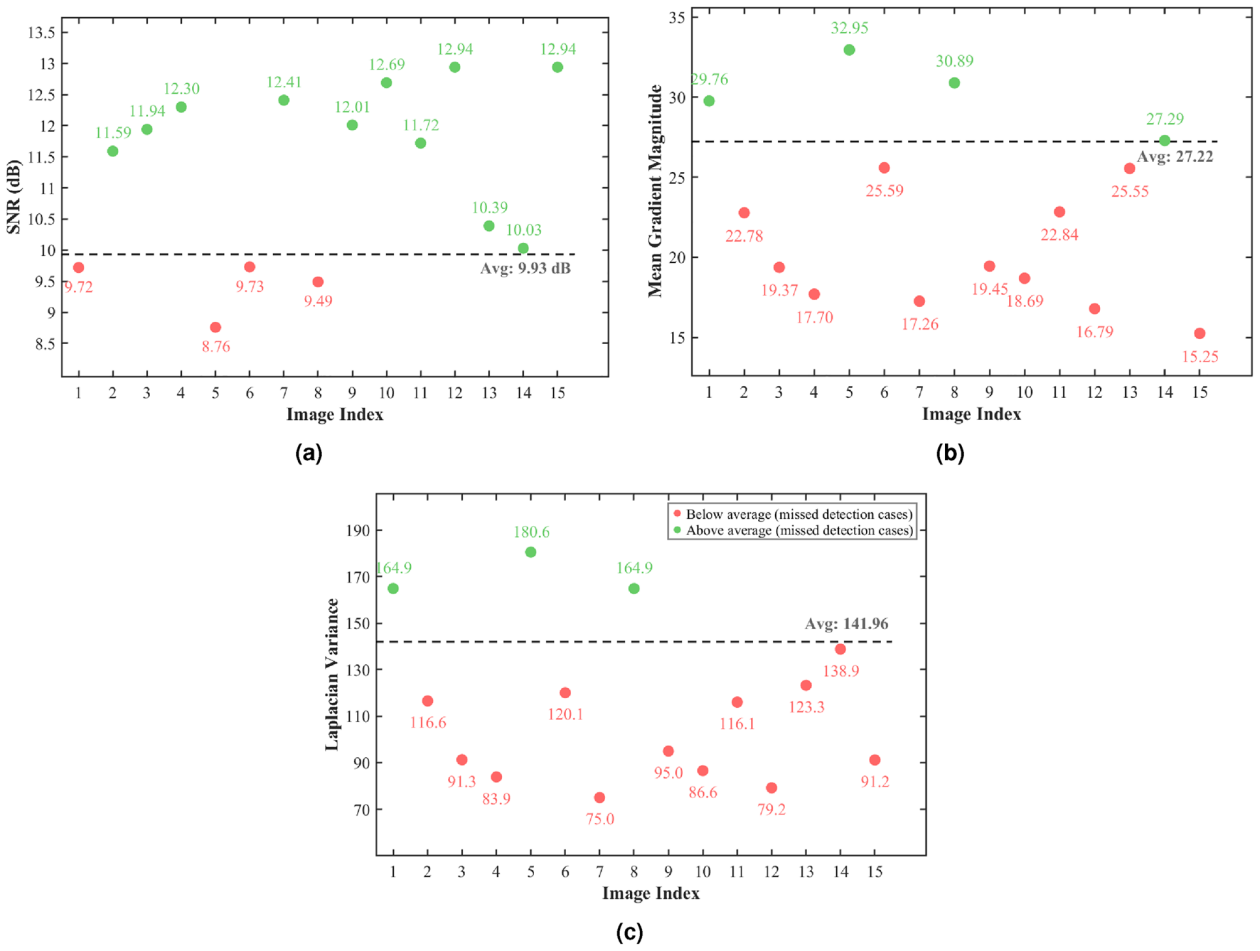
Distribution of image quality metrics for missed detection cases: signal-to-noise ratio, mean gradient magnitude, and Laplacian variance (in sequential order). Dashed lines indicate the average values of the validation set.

#### Imaging quality uncertainty

Fluctuations in light source intensity and uniformity, camera sensor noise, and ambient stray light directly affect three key image quality metrics: the signal-to-noise ratio (SNR, quantifying the strength of the image signal relative to noise), the mean gradient magnitude (reflecting edge and texture sharpness), and the Laplacian variance (characterizing local contrast and detail richness). Variations in these metrics essentially quantify physical disturbances in the image data.

To investigate the relationship between physical disturbances and detection performance, we calculated the average values of the three metrics across all images in the validation set and compared them with the corresponding metrics from the 15 images with missed detections (the metrics shown in Fig. 12 are SNR, mean gradient magnitude, and Laplacian variance, respectively). The analysis revealed that all missed detection images exhibited at least one metric below the validation set average. This statistical pattern indicates that the degradation in image quality induced by the physical environment introduces systematic perturbations into the image data input to the deep learning model. These perturbations directly undermine the stability of feature extraction and the reliability of target recognition, thereby constituting a primary physical source of uncertainty in the detection results.

#### Sample condition uncertainty

**Fig. 13:**
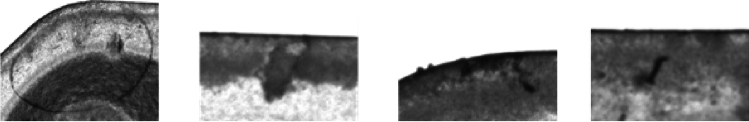
Insert surface contamination example.

As shown in Fig. 13, variations in the surface cleanliness of inserts under actual production conditions cause random reflections and scattering during image acquisition, which compromises the system’s false negative and false positive detection rates.

## Conclusion and outlook

In this study, an automated hardware system for surface defect detection on CNC inserts was designed. To validate the effectiveness of the system, a surface defect dataset was constructed using coated carbide square CNC inserts as the subject. In response to the dataset’s characteristics–such as substantial variation in defect sizes, high intra-class variance, and low inter-class variance–the A2MD-YOLO detection model was proposed. By integrating the CBAM attention mechanism and the P2 detection head, the model effectively enhanced its ability to perceive and focus on defect features. Experimental evaluation demonstrated that the improved model achieved notable gains across four key metrics: precision, recall, $mAP_{50-95}$, and $F_3$. It exhibited particularly strong performance in detecting small defects and classifying complex defect types. The final experimental results indicate that, compared to the baseline YOLOv8 model, A2MD-YOLO increased $mAP_{50-95}$ from 0.529 to 0.571, while the missed detection rate was substantially reduced from 21.4% to 11.8%. Together, these metrics collectively validate the effectiveness of the proposed model improvements. Finally, the model was implemented into the hardware system, enabling automated detection of surface defects on CNC inserts.

Nevertheless, there remains room for further optimization of the proposed system. First, there remains room for improvement in the detection accuracy of the algorithm, particularly in reducing the miss rate for minor defects that are challenging to identify manually. Second, due to the lack of datasets for other types of inserts, the system’s detection performance when handling such inserts requires enhancement; therefore, additional samples of diverse insert types will be collected to establish a comprehensive insert defect dataset repository. Finally, the current average inspection time per insert remains longer than that of manual inspection and does not yet meet the requirements for real-time in-line production. Subsequent efforts will focus on optimizing the hardware configuration to shorten the inspection cycle by reducing the motion time of the robotic arm, with the goal of developing a more efficient and reliable inspection system capable of meeting the requirements for real-time in-line inspection in industrial production.

## Data Availability

The data that support the findings of this study are available from the corresponding author upon reasonable request.
